# Machine Learning Models to Predict In-Hospital Mortality among Inpatients with COVID-19: Underestimation and Overestimation Bias Analysis in Subgroup Populations

**DOI:** 10.1155/2022/1644910

**Published:** 2022-06-23

**Authors:** Javad Zarei, Amir Jamshidnezhad, Maryam Haddadzadeh Shoushtari, Ali Mohammad Hadianfard, Maria Cheraghi, Abbas Sheikhtaheri

**Affiliations:** ^1^Department of Health Information Technology, School of Allied Medical Sciences, Ahvaz Jundishapur University of Medical Sciences, Ahvaz, Iran; ^2^Air Pollution and Respiratory Diseases Research Center, Ahvaz Jundishapur University of Medical Sciences, Ahvaz, Iran; ^3^Social Determinant of Health Research Center, Department of Public Health, School of Health, Ahvaz Jundishapur University of Medical Sciences, Ahvaz, Iran; ^4^Department of Health Information Management, School of Health Management and Information Sciences, Iran University of Medical Sciences, Tehran, Iran

## Abstract

Prediction of the death among COVID-19 patients can help healthcare providers manage the patients better. We aimed to develop machine learning models to predict in-hospital death among these patients. We developed different models using different feature sets and datasets developed using the data balancing method. We used demographic and clinical data from a multicenter COVID-19 registry. We extracted 10,657 records for confirmed patients with PCR or CT scans, who were hospitalized at least for 24 hours at the end of March 2021. The death rate was 16.06%. Generally, models with 60 and 40 features performed better. Among the 240 models, the C5 models with 60 and 40 features performed well. The C5 model with 60 features outperformed the rest based on all evaluation metrics; however, in external validation, C5 with 32 features performed better. This model had high accuracy (91.18%), F-score (0.916), Area under the Curve (0.96), sensitivity (94.2%), and specificity (88%). The model suggested in this study uses simple and available data and can be applied to predict death among COVID-19 patients. Furthermore, we concluded that machine learning models may perform differently in different subpopulations in terms of gender and age groups.

## 1. Introduction

In spite of more than 2 years since the COVID-19 pandemic and performing vaccination in many countries, the disease's prevalence and mortality have not slowed down, and many countries are still experiencing high peaks [1]. In addition, multiple mutations in the virus have become a new challenge to control the disease, leading to the spread of the disease and increased mortality [2–4]. Until April 16, 2022, more than 500 million cases of the disease and more than 6 million deaths due to COVID-19 have been reported globally, with more than 7 million cases and 140,000 deaths in Iran [1].

Since the beginning of the COVID-19 pandemic, one of the most critical challenges for the healthcare systems has been to increase the number of patients with severe symptoms and the growing demand for hospitalization. In developing countries, which do not have sufficient healthcare infrastructure, the increase in inpatients has put a lot of burden on the healthcare system. Moreover, numerous studies have reported various risk factors such as old age, male gender, and underlying medical conditions (such as hypertension, cardiovascular disease, diabetes, COPD, cancer, and obesity) for the deterioration of COVID-19 patients [5–9].

The use of modern and noninvasive methods to triage patients into specific and known categories at the early stages of the disease is beneficial [10]. One of these approaches is the use of predictive models based on machine learning [11, 12]. For example, developing predictive models based on mortality risk factors can positively prevent mortality through controlling acute conditions and planning in intensive care units [13]. Furthermore, machine learning can classify patients based on the deteriorating risk and predict the likelihood of death to manage resources optimally [14, 15].

To date, several studies have been published on the application of machine learning to develop diagnostic models or predict the death of patients due to COVID-19 [14–23]. For example, several deep learning models have been reported to diagnose COVID-19 based on images [24]. In a study, researchers developed an enhanced fuzzy-based deep learning model to differentiate between COVID-19 and infectious pneumonia (no-COVID-19) based on portable CXRs and achieved up to 81% accuracy. Their fuzzy model had only three misclassifications on the validation dataset [24].

As for death prediction, several studies have also been published [16, 25–28]. The results obtained from the studies on machine learning-based predictive methods indicated that those methods had reliable predictability and could identify the correlation between intervening variables in complex and ambiguous conditions caused by COVID-19. Therefore, they can be used to predict such situations in the future. Although those techniques have been tested on some regional datasets of the risk factors, the performance of the models can be improved when they apply to different datasets related to other countries such as Iran, where the prevalence of the COVID-19 and related deaths is high.

Iran is one of the first countries to face a widespread outbreak of the disease and has experienced more than four major epidemic waves with the highest mortality rates [29, 30]. As a result, due to the high prevalence and mortality rate of COVID-19 in Iran and the limitation of healthcare resources [31, 32], it is vital to have a prediction model based on Iranian conditions and local data. Therefore, this study aimed to fit a model for predicting the death caused by COVID-19 based on machine learning algorithms. Many previous models are based on laboratory, imaging, or treatment data [16, 25–28]; however, we suggested models based on available demographic data, symptoms, and comorbidities that can be easily collected. We also conducted a bias analysis of machine learning models based on subgroups of patient populations to show the bias of these models.

## 2. Materials and Methods

### 2.1. Population and Data

We extracted data from the Khuzestan COVID-19 registry system belonging to Ahvaz Jundishapure University of Medical Sciences (AJUMS). From the beginning of the pandemic, this registry collects data from suspected (based on clinical signs) and confirmed (based on the results of PCR or CT scan) outpatients and inpatients in Khuzestan province, Iran. This registry collects demographic data, signs and symptoms, patient outcomes, PCR and CT results, and comorbidities from 38 hospitals. The details of data collection and data quality control were published elsewhere [30].

**Figure 1:**
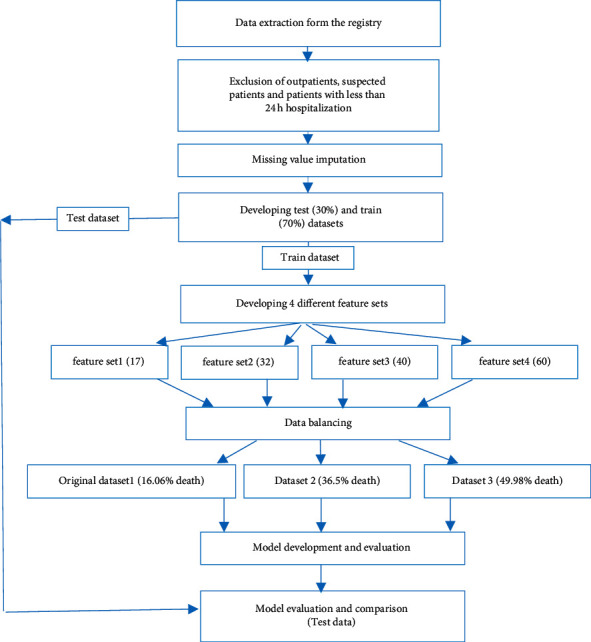
Overview of the study steps.

We included only patients with a confirmed diagnosis of COVID-19 based on PCR test or CT scan results for this modeling study. Furthermore, we included only patients who were hospitalized for more than 24 hours. Because outpatients and hospitalized patients with a short stay (less than 24 hours) had a lot of missing data, we excluded these cases from the final analysis. We also included patients from all age groups. Finally, we extracted data for 10,657 patients. The frequency of nonsurviving patients (until discharge) was 1711 (16.06%); 8946 patients (83.94%) were discharged alive.  Figure 1 shows the steps of this study.

### 2.2. Data Preprocessing

#### 2.2.1. Imputing Missing Variables

Because of the data quality controls in the registry, the database had a low rate of missing data. The 28 variables had a missing rate below 4% (Supplement 1, Table S1). In machine learning, data imputation is a standard approach to improve the models' performance. Different methods such as imputation with mean, median, or mode are common. We imputed the missing values with the mean for age and the highest frequency of values for nonnumerical variables as well [11, 33].

#### 2.2.2. Features and Feature Selection

The outcome measure of the study is in-hospital mortality until discharge which is collected as binary (yes/no). The dataset contains 60 input variables. Age and the number of comorbidities are numerical; oxygen saturation level (PO2) includes two values including below and above 93%. We created three dummy variables for the diagnosis method (only positive PCR, only abnormal CT, positive PCR, and abnormal CT). Other variables have two values: yes or no.

For feature selection, we applied univariate analysis using Chi-square or Fisher exact tests for nonnumerical variables and Mann-Whitney *U* test for age and number of comorbidities (due to abnormal distribution). We created different feature sets to build the prediction models. The first set included all the 60 variables. The second set consisted of variables that were significant in univariate analysis (*P* value <0.05). The third feature set included the marginal variables based on univariate analysis (*P* value <0.2). To create the fourth feature set, we used the feature selection node in the IBM SPSS modeler. This node identifies important features based on univariate analysis as well as the frequency of missing values and the percentage of records with the same value.  Table 1 shows the variables in each of these feature sets.

#### 2.2.3. Data Balancing

We first developed our models with a variety of machine learning algorithms on the original dataset (dataset 1). We found the inappropriate performance of these models, in terms of the sensitivity, because of the small number of samples in the death class (83.94% surviving vs. 16.06% nonsurviving, ratio = 5.23), so the models did not perform well to predict death. There are various methods such as oversampling the minor class or undersampling the major class to solve this problem [11, 12]. We oversampled the death cases to create more balanced datasets. Datasets 2 and 3 included 5,133 (36.5%, ratio = 1.74) and 8,938 (49.98%, ratio = 1) nonsurviving patients, respectively. We developed our models with all four feature sets on these three datasets.

### 2.3. Model Development and Evaluation

We randomly divided the data into two sets, training (70%) and testing (30%) sets, and developed our models using common machine learning algorithms that are usually reported to perform well in medicine including Multiple Layer Perceptron (MLP) neural networks [11, 12, 34], Chi-Squared Detection of Automatic Interaction (CHAID), C5, and Random Forest (RF) decision trees [11, 12, 33, 34], Support Vector Machine (SVM) with Radial Basic Function (RBF) kernel [12, 35, 36], and Bayesian network [12, 37–39].

We first developed models based on the default settings of parameters. We developed CHAID decision trees with a maximum depth of five and a minimum record of two in the nodes. Moreover, we implemented the C5 tree with a minimum of two records in nodes. RF was also implemented with a maximum depth of 10, and a minimum of five records in nodes using 100 models. The SVM model was implemented with a regularization parameter of 10 and a gamma of 0.1. We additionally developed MLPs using the different number of neurons (5, 10, 15, and 20) in one and two hidden layers and also with the number of neurons suggested by the software. We also implemented the best CHAID, C5, and MLP with boosting ensemble method and 10-fold cross-validation. Furthermore, we implemented stack models (combining individual models) [40]. Our analysis showed that models developed on dataset 3 had generally better performance. Therefore, we developed stack models, based on the best individual models, on this dataset with different feature sets.

### 2.4. External Validation

For external validation, we extracted 1734 records from the Khuzestan COVID-19 registry system. These data are from four different hospitals in different timeframes. Therefore, these data were not used in training or testing the models. This dataset contained 1425 surviving and 309 nonsurviving patients. Inclusion and exclusion criteria were similar to the training/testing dataset, described in Section 2.1. The best performing models selected from the previous step and also ensemble models were validated using this dataset.

### 2.5. Subpopulation Bias Analysis

Previous studies show that predictive models may have different performances against different subpopulations, for example, in different sex or age groups [41, 42]. To assess this effect, we adopted the method suggested by Seyyed-Kalantari et al. They suggested the use of false-positive rate (FPR) and false-negative rate (FNR) in subpopulations to assess the underdiagnosis and overdiagnosis of machine learning models [41]. We similarly calculated FNR and FPR to assess the underprediction or overprediction of death in our models. To this end, we used the best performing models in external evaluation and the external dataset.

### 2.6. Analysis

We applied IBM SPSS statistical software version 23 for statistical analysis and IBM SPSS modeler version 18 to develop and evaluate machine learning models. We evaluated and compared the models using confusion matrix, accuracy, precision, sensitivity, specificity, F-score, and Area under the Curve (AUC). To select the best performing models, we compared the models obtained from each dataset-feature with each other based on AUC and F-score.

### 2.7. Ethical Considerations

This study received ethical approvals from the Ethics Research Committee of Ahvaz Jundishapur University of Medical Sciences (IR.AJUMS.REC.1400.325).

## 3. Results

### 3.1. Descriptive Data

We extracted data for 10,657 patients from the Khuzestan COVID-19 registry [30]. The frequency of nonsurviving patients (until discharge) was 1711 (16.06%); 8946 patients (83.94%) were discharged alive.  Table 2 shows that the death due to COVID-19 was significantly higher among men, older patients, and those who have been in contact with infected individuals. In addition, respiratory distress, convulsion, altered consciousness, and paralysis were more common among the nonsurviving patients. Conversely, cough, headache, diarrhea, and dizziness were less prevalent among them. Furthermore, oxygen saturation status was better among the recovered patients versus the dead. Moreover, the comorbidities and risk factors (excluding pregnancy) as well as the intubation, oxygen therapy at the beginning of hospitalization, and ICU admission were significantly higher among the dead.

### 3.2. The Machine Learning Algorithms and Their Evaluation

The results of performing various models with different settings on three datasets and four feature groups are reported as follows.

#### 3.2.1. The Machine Learning Algorithms on Original Dataset 1

The details on the performance of the models are given in Supplement 1 (Tables S2–S5). The result showed that the lowest and highest accuracy of the models based on the original dataset 1 were 84.52% (RF with 32 features) and 91.12% (Bayesian network with 32 features), respectively. In addition, the minimum and maximum AUC were 0.757 (C5 with 32 features) and 0.914 (Bayesian network with 32 features), respectively. According to the findings, the sensitivity for predicting death based on original dataset 1 was low and between 0.484 (MLP network with 60 features) and 0.775 (RF with 32 features) which indicates that the sensitivity of the models on imbalanced data is not appropriate.  Table 3 shows the results of the performance of the top 10 models based on the test data of dataset 1. According to the table, the best two models were the Bayesian network and the CHAID tree on 32 features, respectively. The ROC curve for the best models is presented in Supplementary Figure S1.

#### 3.2.2. The Machine Learning Algorithms on Dataset 2

The details on the performance of the models based on dataset 2 are given in Supplement 1, Tables S6–S9. The findings showed that the lowest and highest accuracy were 82.64% (MLP with 60 features) and 87.86% (RF with 60 features), respectively. Moreover, the minimum and maximum values of the AUC were 0.888 (MLP with 60 features) and 0.942 (SVM with 60 features), respectively. According to the findings, the sensitivity for predicting death was between 0.658 (MLP network) and 0.861 (CHAID tree with 32 features). The best results obtained for each algorithm based on dataset 2 were shown in Supplementary Figure S2. According to Table 4, SVM and C5 models had the best performance on 60 and 40 features, respectively.

#### 3.2.3. The Machine Learning Algorithms on Dataset 3

The details on the performance of the models based on dataset 3 are given in Supplement 1, Tables S10–S13. The results showed that the lowest and highest accuracy were 81.27% (CHIAD tree with 32 features) and 92.77% (C5 with 60 features), respectively. Moreover, the minimum and maximum AUC were 0.899 (CHIAD with 32 features) and 0.972 (C5 with 60 features), respectively. The sensitivity for predicting death was also between 0.752 (MLP with 60 features) and 0.951 (C5 tree with 60 features). The best results obtained for each algorithm based on dataset 3 are shown in Supplementary Figure S3. According to Table 5, the C5 model had the best performance with different features, and SVM with 60 features was also one of the optimal models.

### 3.3. Ensemble Models

Table 6 indicates that the best ensemble model had 89.13% accuracy and 0.961 AUC. However, the comparison of these models with the corresponding individual models (Table 5) shows that C5 models have better performance than these ensemble models, even though these ensemble models are better than other individual models.

### 3.4. External Validation

We evaluated all ensemble models (Table 6) and the top 10 models developed on dataset 3 (Table 5) using an external dataset. As shown in Table 7, C5 boosting models with feature sets 1 and 2 have better scores.

### 3.5. Subpopulation Bias Analysis

**Figure 2:**
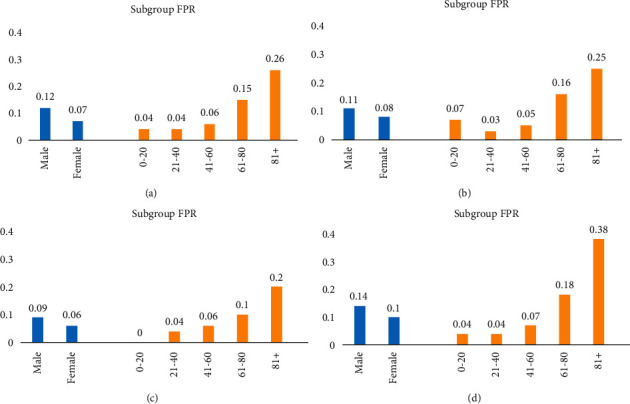
Subgroup false-positive rate (FPR) for different models. (a) C5 model on feature set 1. (b) C5 model on feature set 2. (c) SVM model on feature set 3. (d) Ensemble model on feature set 2.

**Figure 3:**
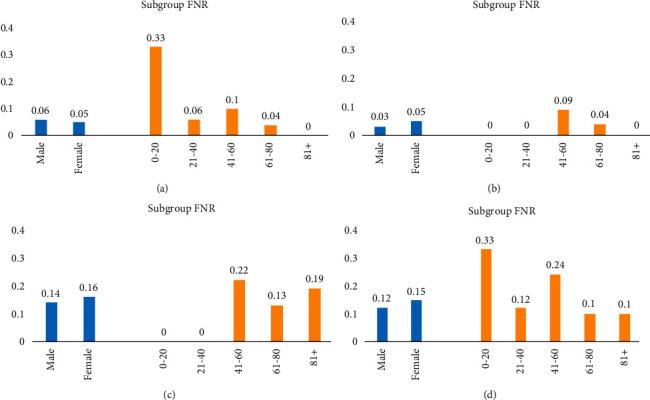
Subgroup false-negative rate (FNR) for different models. (a) C5 model on feature set 1. (b) C5 model on feature set 2. (c) SVM model on feature set 3. (d) Ensemble model on feature set 2.

We selected the four best models based on external validation for subpopulation bias analysis (Supplement 1, Table S14). Figures 2 and 3 show the FPR and FNR of these models. As these figures indicate, most of these models better perform on female patients than male patients. Furthermore, the performance of these models decreases in older patients. As for FPR, Figure 2 indicates that SVM and C5 (feature set 2) have a less biased prediction in terms of gender and age groups. Additionally, Figure 3 shows that C5 (feature set 2) has a less biased prediction.

### 3.6. Comparison of the Models

A comparison of the models showed that, with the balancing of the data, the sensitivity and AUC increased. However, the accuracy based on dataset 2 decreased, but it also increased based on dataset 3. Furthermore, models with 60 and 40 features performed better. In general, the C5 model with 60 features outperformed the rest based on all evaluation indicators; however, based on the external validation, C5 boosting models with feature sets 1 (17 features) and 2 (32 features) have better external validity. Subpopulation analysis suggests that the C5 boosting model with 32 features has less bias.

### 3.7. Variable Importance

**Figure 4:**
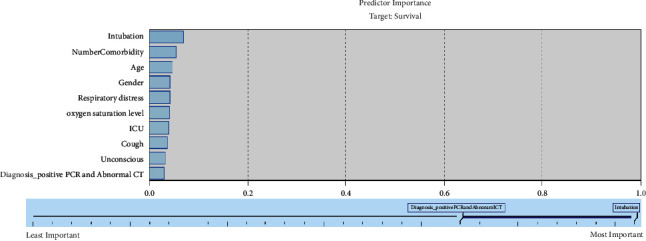
Variable importance of the selected model.

Figure 4 shows the importance of each variable in the selected model (C5). As indicated, intubation, number of comorbidities, age, gender, respiratory distress, blood oxygen saturation level, ICU admission, cough, unconsciousness, positive PCR, and abnormal CT are considered the most important death predictors by this model.

## 4. Discussion

In the first stage of the study, the risk factors for death due to COVID-19 were discovered using univariate analysis. Then, based on the important features, different machine learning models were developed to predict death. The results showed significant differences between recovered and nonrecovered patients in terms of age, sex, contact with infected people, respiratory distress, convulsion, altered consciousness, paralysis, blood oxygen saturation level, the number of comorbidities, intubation, oxygen therapy, and the need for ICU services.

We found that intubation, number of comorbidities, age, gender, respiratory distress, blood oxygen saturation level, ICU admission, cough, unconsciousness, positive PCR, and abnormal CT are the most important death predictors. Other studies showed that age [17, 18, 23, 27, 28, 43], male gender [43], respiratory disease [16, 17], the number of comorbidities [43], and low oxygen saturation [17, 18, 23, 43] increased cases of death due to COVID-19. Some researchers indicate that high blood pressure, heart disease, cancer, kidney disease [16, 17], diabetes [18], cerebrovascular diseases [28], smoking [18, 23], and asthma [16] increased mortality from COVID-19. However, our model did not consider these factors significant. It is worth mentioning that these risk factors increased the number of comorbidities in a patient and this factor was also considered significant in the C5 model.

We developed various models with different features to predict death from COVID-19. Based on the results, the best performance was related to the C5 decision tree with 32 features. In the same way, several studies tried to develop machine learning models for predicting death from COVID-19 [16–23, 25–28, 43–45]. Since a variety of variables (demographic, laboratory, radiographic, therapeutic, signs and symptoms, and comorbidities) and datasets are used, it is not easy to compare the studies. For example, some researchers used laboratory data to develop models in addition to other variables [17, 23, 28, 43], and a study applied only laboratory variables [45]. In another study, vital signs and imaging results were used to develop models [23]. However, the variables used in our study were similar to most of the studies. Despite this, a comparison of our study with previous studies showed that the performance of our selected model was better than those models (Table 8). The model developed by Gao et al. [43] has better performance (AUC = 0.976 vs. AUC = 0.972); however, this model was developed with small sample size. In addition, the F-score (*F* = 0.97) of the model developed by Yan et al. [19] was higher than our selected model. However, Barish et al. [46] showed that Yan's model did not have a good result in the external validation. Khan's model [26] also has a higher F-score than our model. Khan et al. and Gao et al. used unbalanced data; Barish et al. [46] have shown that models developed based on unbalanced data to predict death from COVID-19 may not have accurate results in the real environment.

We found that machine learning models perform differently in subpopulations in terms of gender and age groups. Other studies similarly show that predictive models have different performances in different ethnic groups, genders, and age groups of patients and patients with different insurance [41, 42]. Therefore, researchers and clinicians should apply these models to different population groups cautiously. Moreover, developing models for different patient groups may be necessary.

The strengths of our model are the use of demographic data, symptoms, and comorbidities that can be easily collected. Despite some previous studies, we did not use laboratory, treatment, and imaging data. It can be considered a limitation. However, we supposed that all patients received almost similar treatments. Moreover, applying models which are developed based on treatment data may be difficult because of changes in patients' treatment. Furthermore, models that depend on laboratory and imaging data require a lot of time and cost to gather these data to use the model in a real clinical environment. A comparison of our study with those that used laboratory and imaging data (Table 8) indicates that our selected model outperforms many of these models. A study also indicated that imaging data did not affect the performance of machine learning models to predict death from COVID-19 [23]. In addition, the data used in our study have been collected from 38 hospitals, which is the strength of the study. A similar study indicated that up to 20% of missing data in COVID-19 studies is acceptable for developing machine learning models [18]; however, the missing rate in our study was under 4%.

Despite the strengths, some limitations should be considered. Firstly, we only analyzed the subpopulation bias based on gender and age groups. Future studies should consider other variables in this analysis. Furthermore, there are several well-established models such as APACHE and SOFA [41, 42]. Researchers are recommended to compare the performance of machine learning models with these models to predict deaths from COVID-19.

## 5. Conclusions

Different machine learning models were developed to predict the likelihood of death caused by COVID-19. The best prediction model was the C5 decision tree (accuracy = 91.18%, AUC = 0.96, and *F* = 0.916). Therefore, this model can be used to detect high-risk patients and improve the use of facilities, equipment, and medical practitioners for patients with COVID-19.

## Figures and Tables

**Table 1:** Different feature sets.

Feature set	Method	Number of features	Features
1	Feature selection node (default setting)	17	Age, contact with COVID-19 patients, cough, diabetes, diagnosis only by abnormal CT, diagnosis only by positive PCR, diagnosis by positive PCR and abnormal CT, gender, heart diseases, HTN, and ICU. Admission, intubation, muscle ache, number of comorbidity, oxygen therapy blood oxygen saturation level, and respiratory distress.

2	Univariate analysis (*P* value <0.05)	32	Age, cancer, chronic kidney disease, chronic liver disease, contact (with a probable or confirmed case in the 14 days before the onset of symptoms), convulsion, cough, diabetes, diagnosis only by abnormal CT, diagnosis only by positive PCR, diagnosis by positive PCR and abnormal CT, dialysis, diarrhea, dizziness, drug abuse, gender, headache, heart diseases, HIV/AIDS, HTN, and ICU. Admission, immune diseases, intubation, nervous system diseases, number of comorbidities, other chronic lung diseases, oxygen therapy, paralysis, blood oxygen saturation level, pregnancy, respiratory distress, and unconsciousness.

3	Univariate analysis (*P* value <0.2)	40	The feature set 2 + asthma, chronic hematology diseases, mental disorders, muscle ache, other diseases (comorbidities), drowsiness, gustatory dysfunction, and weakness.

4	All features	60	The feature set 3 + abdominal pain, autoimmune disease, chest pain, chills, constipation, ocular manifestations, fever, GI bleeding, hemoptysis, nausea, anorexia, other GI signs, paresis, runny nose, skin manifestations, sore throat, olfactory dysfunction, smoking, sweating, and vomiting.

**Table 2:** Comparison of surviving and nonsurviving patients.

Variables	Alive (*n* = 8946)	Dead (*n* = 1711)	Total patients (*n* = 10657)	*P* value
*Age*				
Mean (±SD), years	54 ± 18.3	65.7 ± 16.2	55.88 ± 18.46	<0.0001^*∗*^
Median (Q1, Q3)	56 (42, 67)	67 (57, 77)	58 (43, 69)	
Sex, male	4611 (51.5)	1010 (59)	5621 (52.7)	<0.0001^*∗*^
Contact with infected people (yes)	3169 (35.4)	706 (41.3)	3875 (36.4)	<0.0001^*∗*^
*Sign and symptoms*				
Cough (yes)	5296 (59.2)	899 (52.5)	6195 (58.1)	<0.0001^*∗*^
Respiratory distress (yes)	5021 (56.1)	1288 (75.3)	6309 (59.2)	<0.0001^*∗*^
Fever (yes)	4225 (47.2)	802 (46.9)	5027 (47.2)	0.788
Muscle aches (yes)	2417 (27)	426 (24.9)	2843 (26.7)	0.069
Chills (yes)	70 (0.8)	9 (0.5)	79 (0.7)	0.257
Vomiting (yes)	452 (5.1)	79 (4.9)	531 (5)	0.448
Headache (yes)	480 (5.4)	51 (3)	531 (5)	<0.0001^*∗*^
Chest pain (yes)	304 (3.4)	61 (3.6)	365 (3.4)	0.728
Diarrhea (yes)	315 (3.5)	40 (2.3)	355 (3.3)	0.012^∗^
Sore throat (yes)	48 (0.2)	4 (0.2)	52 (0.5)	0.100
Gustatory dysfunction (yes)	98 (1.1)	10 (0.6)	108 (1)	0.053
Olfactory dysfunction (yes)	123 (1.4)	19 (1.1)	142 (1.3)	0.382
Abdominal pain (yes)	203 (2.3)	31 (1.8)	234 (2.2)	0.237
Runny nose (yes)	8 (0.1)	0 (0.0)	8 (0.1)	0.216
Convulsion (yes)	42 (0.5)	19 (1.1)	61 (0.6)	0.001^*∗*^
Altered consciousness (yes)	213 (2.4)	419 (24.5)	633 (5.9)	<0.0001^*∗*^
GI bleeding (yes)	5 (0.1)	0 (0.0)	5 (0.0)	0.417
Skin lesion/rush (yes)	11 (0.1)	3 (0.2)	14 (0.1)	0.584
Dizziness (yes)	249 (2.8)	30 (1.8)	279 (2.6)	0.014^*∗*^
Paresis (yes)	54 (0.6)	11 (0.6)	65 (0.6)	0.848
Paralysis (yes)	22 (0.2)	13 (0.8)	35 (0.3)	0.001^*∗*^
Weakness (yes)	350 (3.9)	80 (4.7)	430 (4)	0.142
Sweating (yes)	11 (0.1)	2 (0.1)	13 (0.1)	0.947
Ocular manifestations (yes)	3 (0.0)	0 (0.0)	3 (0.0)	0.449
Hemoptysis (yes)	6 (0.1)	2 (0.1)	8 (0.1)	0.491
Drowsiness (yes)	3 (0.0)	2 (0.1)	5 (0.0)	0.185
Constipation (yes)	7 (0.1)	1 (0.1)	8 (0.1)	0.784
Nausea (yes)	478 (5.3)	89 (5.2)	567 (5.3)	0.811
Anorexia (yes)	724 (8.1)	138 (8.1)	862 (8.1)	0.969
Other GI symptoms (yes)	7 (0.1)	0 (0.0)	7 (0.1)	0.247
*Blood oxygen saturation level*				
(i) Less than 93	2046 (22.9)	934 (54.6)	2980 (28)	<0.0001^*∗*^
(ii) More than 93	6900 (77.1)	777 (45.4)	7677 (72)	
*Comorbidity*				
Any comorbidity (yes)	3314 (37)	826 (48.3)	4140 (38.8)	<0.0001^*∗*^
Number of comorbidities				<0.0001^*∗*^
0	5632 (63)	885 (51.7)	6517 (61.2)	
1	1868 (2.9)	391 (22.9)	2259 (21.2)	
2	946 (10.6)	275 (16.1)	1221 (11.5)	
3	396 (4.4)	112 (6.5)	508 (4.8)	
>3	104 (1.1)	48 (2.8)	152 (1.5)	
Number of comorbidities (mean ± SD)	0.6 ± 0.9	0.87 ± 1.1	0.65 ± 0.97	<0.0001^*∗*^
Hypertension (yes)	1291 (14.4)	356 (20.8)	1647 (5.5)	<0.0001^∗^
Heart diseases (yes)	1102 (12.3)	294 (17.2)	1396 (13.11)	<0.0001^*∗*^
Diabetes (yes)	1577 (17.6)	376 (22)	1953 (18.3)	<0.0001^*∗*^
Immunodeficiency diseases (yes)	32 (0.4)	13 (0.8)	45 (0.4)	0.019^*∗*^
Asthma (yes)	198 (2.2)	28 (1.6)	226 (2.1)	0.129
Neurological diseases (yes)	140 (1.6)	49 (2.9)	189 (1.8)	<0.0001^*∗*^
Chronic kidney diseases (yes)	289 (3.2)	114 (6.7)	403 (3.8)	<0.0001^*∗*^
Dialysis (yes)	78 (0.9)	33 (1.9)	111 (1)	<0.0001^*∗*^
Other chronic lung diseases (yes)	136 (1.5)	44 (2.6)	180 (1.7)	0.002^*∗*^
Chronic hematologic diseases (yes)	740 (0.8)	20 (1.2)	94 (0.9)	0.166
Cancer (yes)	172 (1.9)	80 (4.7)	252 (2.4)	<0.0001^*∗*^
Autoimmune diseases (yes)	2 (0.0)	0 (0.0)	2 (0.0)	0.536
Chronic liver diseases (yes)	46 (0.5)	16 (0.9)	62 (0.6)	0.036^*∗*^
HIV/AIDS (yes)	7 (0.1)	5 (0.3)	12 (0.1)	0.016^*∗*^
Mental disorders (yes)	26 (0.3)	2 (0.1)	28 (0.3)	0.198
Smoking (yes)	143 (1.6)	33 (1.9)	176 (1.7)	0.326
Drug abuse (yes)	54 (0.6)	21 (1.2)	75 (0.7)	0.005^∗^
Other comorbidities (yes)	286 (3.2)	69 (4)	355 (0.0)	0.078
Pregnancy	63 (0.7)	2 (0.1)	65 (0.6)	0.004^*∗*^
*Care and treatment*				
Intubation (yes)	308 (3.44)	962 (56.2)	1270 (11.9)	<0.0001^*∗*^
ICU care (yes)	1323 (14.8)	1088 (63.6)	2411 (22.6)	<0.0001^*∗*^
Oxygen therapy (yes)	2921 (32.7)	682 (39.9)	3603 (33.8)	<0.0001^*∗*^
*Diagnosis method*				
(i) Only abnormal CT	3197 (35.7)	583 (31.4)	3735 (35)	<0.0001^*∗*^
(ii) Only positive PCR	1161 (13)	160 (9.4)	1321 (12.4)	<0.0001^*∗*^
(iii) Positive PCR and abnormal CT	4588 (51.3)	1013 (59.2)	5601 (52.6)	<0.0001^*∗*^

^*∗*^Significant difference.

**Table 3:** Top 10 models developed on original dataset 1.

	Setting	Feature set	Accuracy	Sensitivity	Specificity	Precision	F-score	AUC
Bayesian network	Default	2	91.12	64.7	96.2	76.4	0.701	0.914
CHIAD	Default	2	90.76	54	97.8	82.6	0.653	0.909
MLP^*∗*^	2.5.5 boosting	1	90.63	53.6	97.7	81.5	0.647	0.904
MLP	Boosting 1.10	3	90.79	54	97.8	82.3	0.652	0.903
C5	Boosting	2	90.7	56.4	97.3	79.9	0.662	0.901
MLP	2.10.10	2	90.55	53.4	97.7	81.5	0.646	0.901
MLP	2.5.5	1	90.31	55.4	97	77.6	0.646	0.901
RF	Default	2	84.52	77.5	85.9	51.3	0.617	0.9
MLP	2.20.20	3	90.51	53.6	97.5	80.5	0.643	0.899
Bayesian network	Default	1	90.46	55.5	97.1	78.5	0.65	0.899

^*∗*^For MLPs, the numbers for MLP indicate the number of layers, the number of neurons in hidden layer 1, and the number of neurons in hidden layer 2.

**Table 4:** Top 10 models developed on dataset 2.

	Settings	Feature set	Accuracy	Sensitivity	Specificity	Precision	F-score	AUC
SVM	RBF default	4	87.83	83.4	90.3	82.9	0.832	0.942
C5	Boosting	3	87.44	81.8	90.6	82.7	0.822	0.94
SVM	RBF default	3	87.59	82.7	90.3	82.4	0.826	0.938
C5	Boosting	4	87.88	79.9	92.4	85.5	0.826	0.938
RF	Default	4	87.86	85.7	89.1	81.5	0.836	0.931
C5	Boosting	2	86.68	78.5	91.5	84.3	0.813	0.927
C5	Boosting	1	85.99	77.2	90.8	82.2	0.797	0.926
SVM	RBF default	2	86.61	79	91.1	83.7	0.813	0.926
MLP^*∗*^	1.10	3	85.38	77	90	80.9	0.789	0.923
RF	Default	1	85.26	85.2	85.3	76.2	0.804	0.923

^*∗*^For MLPs, the numbers for MLP indicate the number of layers, the number of neurons in hidden layer 1, and the number of neurons in hidden layer 2.

**Table 5:** Top 10 models developed on dataset 3.

	Settings	Feature set	Accuracy	Sensitivity	Specificity	Precision	F-score	AUC
C5	Boosting	4	92.77	95.1	90.5	90.8	0.929	0.972
C5	Boosting	3	91.74	93.6	89.8	90.5	0.92	0.965
C5	Boosting	2	91.18	94.2	88	89.1	0.916	0.96
SVM	RBF default	4	90.16	92.7	87.7	88.1	0.903	0.956
C5	Boosting	1	89.28	91.3	87.3	87.7	0.895	0.952
SVM	RBF default	3	88.81	90.5	87.1	87.9	0.892	0.944
MLP^*∗*^	2.15.15 boosting	3	88.59	90.2	86.9	87.7	0.889	0.94
MLP	2.12.12 boosting	4	87.61	88.5	86.8	86.8	0.876	0.938
C5	Default	3	87.4	89.8	85	86.1	0.879	0.934
SVM	RBF default	2	86.34	86.6	86.1	86.6	0.866	0.932

^*∗*^For MLPs, the numbers for MLP indicate the number of layers, the number of neurons in hidden layer 1, and the number of neurons in hidden layer 2.

**Table 6:** Ensemble models developed on dataset 3.

ID	Included models	Feature set	Accuracy	Sensitivity	Specificity	Precision	F-score	AUC
1	Table S10	1	86.10	0.799	0.924	0.914	0.853	0.954
2	Table S11	2	87.39	0.859	0.889	0.888	0.873	0.954
3	Table S12	3	87.26	0.831	0.915	0.908	0.867	0.954
4	Table S13	4	89.13	0.864	0.919	0.916	0.890	0.961

**Table 7:** External validation on dataset 3.

Models	Settings	Feature set	Accuracy	Sensitivity	Specificity	Precision	F-score	AUC
C5	Boosting	1	92.56	0.955	0.919	0.720	0.821	0.974
C5	Boosting	2	91.81	0.964	0.908	0.695	0.808	0.98
SVM	RBF default	3	91.00	0.848	0.924	0.706	0.771	0.955
Ensemble 2	—	2	87.77	0.861	0.881	0.611	0.715	0.954
SVM	RBF default	2	88.24	0.890	0.881	0.618	0.729	0.953
Ensemble 1	—	1	88.75	0.819	0.902	0.645	0.722	0.949
C5	Boosting	3	86.51	0.935	0.850	0.575	0.712	0.948
Ensemble 3	—	3	88.18	0.783	0.903	0.637	0.702	0.931
MLP^*∗*^	2.15.15 boosting	3	87.95	0.767	0.904	0.634	0.694	0.914
MLP^*∗*^	2.12.12 boosting	4	87.31	0.754	0.899	0.618	0.679	0.914
Ensemble 4	—	4	86.62	0.770	0.887	0.596	0.672	0.91
C5	Boosting	4	85.64	0.748	0.880	0.575	0.650	0.889
C5	Default	3	85.24	0.780	0.868	0.562	0.653	0.887
SVM	RBF default	4	83.79	0.725	0.862	0.533	0.615	0.868

^*∗*^For MLPs, the numbers for MLP indicate the number of layers, the number of neurons in hidden layer 1, and the number of neurons in hidden layer 2.

**Table 8:** Some machine learning models suggested in the literature to predict death from COVID-19.

Author	Number of patients, death rate, number of features	Models	Accuracy	AUC
Muhammad et al. [44]	1505, NA, 4	Decision tree (DT)	99.85	NA
		LR	97.49	NA
		SVM	98.85	NA
		Naive Bayes	97.52	NA
		RF	99.60	NA
		KNN	98.06	NA
Pourhomayoun and Shakibi [22]	307382, NA, 57	RF	87.93	0.94
		ANN	89.98	0.93
		SVM	89.02	0.88
		KNN	89.83	0.90
		LR	87.91	0.92
		DT	86.87	0.93
Li et al. [20]	2924, 8.8%, different features (83, 152, 5)	Gradient boosting decision tree, 83 features	88.9	0.939
		LR, 152 features	86.8	0.928
		LR, 5 features	88.7	0.915
Goncalves and Rouco [21]	827601, 8.7%, 3	Adaboost, gradient boosting, and RF	NA	0.919
		LR	NA	0.917
An et al. [16]	8000, 2.2%, 10	SVM linear	91.9	0.962
		LASSO	91.1	0.963
		LASSO (14 days)	86.8	0.944
		SVM linear (14 days)	87.7	0.941
		LASSO (30 days)	89.5	0.953
		SVM linear (30 days)	87.7	0.948
Yadaw et al. [18]	3841, 8.1%, 17 and 3	XGBoost (17 and 3 features)	NA	0.91
Yan et al. [19]	375, 35%, 3	XGBoost	90	F1: 0.97^∗^
Gao et al. [43]	2160, 11%, 14	SVM	95.8	0.976
		ANN	95.6	0.976
		Ensemble	95.5	0.976
		LR	95.4	0.974
		GBDT	94.8	0.953
Chen et al. [28]	(192, 26%) only critically ill patients, 47 (17 nonlaboratory, 30 laboratory)	SVM linear	93 (47 features) 87.8 (17 features) 85.6 (30 features)	NA
Booth et al. [45]	398, 10.8%, 5	SVM-RBF		93
Parchure et al. [17]	567, 17.8%, 55	RF	65.5	85.5
Zhao et al. [23]	641, 12.8%, 47	LR	NA	0.82
Das et al. [27]	3524, 2.1%, 4	LR	96.5	0.83
		SVM	97	0.825
		KNN	92.4	0.759
		RF	92.4	0.787
		Gradient boosting	97.1	0.787
Chen et al. [25]	1002 severe and critical cases, 16.1%, 7	LR	NA	0.903
Khan et al. [26]	103888, 5.7%, 15	Deep neural network	0.970	F1: 0.985^*∗*^
		RF, XGBoost	0.946	0.972
		LR, DT	0.945	0.972
		KNN	0.944	0.971

^*∗*^These studies did not report the AUC.

## Data Availability

The data used to support the findings of this study are restricted by the Ethics Research Committee of Ahvaz Jundishapur University of Medical Sciences in order to protect patient privacy.

## Supplementary Materials

Supplementary Materials Supplement 1: detailed Tables S1–S14. Supplement 2: Figures S1–S3.

## References

[1] World Health Organization (2022). WHO coronavirus (COVID-19) dashboard. https://covid19.who.int.

[2] Darby A. C., Hiscox J. A. (2021). Covid-19: variants and vaccination. *BMJ*.

[3] Cosic I., Cosic D., Loncarevic I. (2021). Analysis of mutated SARS-CoV-2 variants using resonant recognition model. *International Journal of Sciences*.

[4] Samaranayake L., Fakhruddin K. S. (2021). SARS-CoV-2 variants and COVID-19: an overview. *Dental Update*.

[5] Malik V. S., Ravindra K., Attri S. V., Bhadada S. K., Singh M. (2020). Higher body mass index is an important risk factor in COVID-19 patients: a systematic review and meta-analysis. *Environmental Science and Pollution Research*.

[6] Parohan M., Yaghoubi S., Seraji A., Javanbakht M., Sarraf P., Djalali M. (2020). Risk factors for mortality in patients with Coronavirus disease 2019 (COVID-19) infection: a systematic review and meta-analysis of observational studies. *The Aging Male*.

[7] Li R. H., Sigurslid H. H. (2020). Predictors of mortality in hospitalized COVID‐19 patients: a systematic review and meta‐analysis.

[8] Mackey K., Ayers C. K., Kondo K. K. (2021). Racial and ethnic disparities in COVID-19-related infections, hospitalizations, and deaths. *Annals of Internal Medicine*.

[9] Li J., Huang D. Q., Zou B. (2021). Epidemiology of COVID‐19: a systematic review and meta‐analysis of clinical characteristics, risk factors, and outcomes. *Journal of Medical Virology*.

[10] Shaban W. M., Rabie A. H., Saleh A. I., Abo-Elsoud M. A. (2020). A new COVID-19 patients detection strategy (CPDS) based on hybrid feature selection and enhanced KNN classifier. *Knowledge-Based Systems*.

[11] Sheikhtaheri A., Orooji A., Pazouki A., Beitollahi M. (2019). A clinical decision support system for predicting the early complications of one-anastomosis gastric bypass surgery. *Obesity Surgery*.

[12] Sheikhtaheri A., Zarkesh M. R., Moradi R., Kermani F. (2021). Prediction of neonatal deaths in NICUs: development and validation of machine learning models. *BMC Medical Informatics and Decision Making*.

[13] Syeda H. B., Syed M., Sexton K. W. (2021). Role of machine learning techniques to tackle the COVID-19 crisis: systematic review. *JMIR medical informatics*.

[14] Pan P., Li Y., Xiao Y. (2020). Prognostic assessment of covid-19 in the intensive care unit by machine learning methods: model development and validation. *Journal of Medical Internet Research*.

[15] Ryan L., Lam C., Mataraso S. (2020). Mortality prediction model for the triage of COVID-19, pneumonia, and mechanically ventilated ICU patients: a retrospective study. *Annals of Medicine and Surgery*.

[16] An C., Lim H., Kim D. W., Chang J. H., Choi Y. J., Kim S. W. (2020). Machine learning prediction for mortality of patients diagnosed with COVID-19: a nationwide Korean cohort study. *Scientific Reports*.

[17] Parchure P., Joshi H., Dharmarajan K. (2020). Development and validation of a machine learning-based prediction model for near-term in-hospital mortality among patients with COVID-19. *BMJ Supportive & Palliative Care*.

[18] Yadaw A. S., Li Y.-c., Bose S., Iyengar R., Bunyavanich S., Pandey G. (2020). Clinical features of COVID-19 mortality: development and validation of a clinical prediction model. *The Lancet Digital Health*.

[19] Yan L., Zhang H.-T., Goncalves J. (2020). An interpretable mortality prediction model for COVID-19 patients. *Nature Machine Intelligence*.

[20] Li S., Lin Y., Zhu T. (2021). Development and external evaluation of predictions models for mortality of COVID-19 patients using machine learning method. *Neural Computing & Applications*.

[21] Goncalves C. P., Rouco J. (2021). Comparing decision tree-based ensemble machine learning models for COVID-19 death probability profiling. *Journal of Vaccines & Vaccination*.

[22] Pourhomayoun M., Shakibi M. (2021). Predicting mortality risk in patients with COVID-19 using machine learning to help medical decision-making. *Smart Health*.

[23] Zhao Z., Chen A., Hou W. (2020). Prediction model and risk scores of ICU admission and mortality in COVID-19. *PLoS One*.

[24] Ieracitano C., Mammone N., Versaci M. (2022). A fuzzy-enhanced deep learning approach for early detection of Covid-19 pneumonia from portable chest X-ray images. *Neurocomputing*.

[25] Chen B., Gu H.-Q., Liu Y. (2021). A model to predict the risk of mortality in severely ill COVID-19 patients. *Computational and Structural Biotechnology Journal*.

[26] Khan I. U., Aslam N., Aljabri M. (2021). Computational intelligence-based model for mortality rate prediction in COVID-19 patients. *International Journal of Environmental Research and Public Health*.

[27] Das A. K., Mishra S., Saraswathy Gopalan S. (2020). Predicting CoVID-19 community mortality risk using machine learning and development of an online prognostic tool. *PeerJ*.

[28] Chen Y., Linli Z., Lei Y. (2020). Risk factors for mortality in critically ill patients with COVID‐19 in Huanggang, China: a single‐center multivariate pattern analysis. *Journal of Medical Virology*.

[29] Ghafari M., Kadivar A., Katzourakis A. (2021). Excess deaths associated with the Iranian COVID-19 epidemic: a province-level analysis. *International Journal of Infectious Diseases*.

[30] Zarei J., Dastoorpoor M., Jamshidnezhad A., Cheraghi M., Sheikhtaheri A. (2021). Regional COVID-19 registry in Khuzestan, Iran: a study protocol and lessons learned from a pilot implementation. *Informatics in Medicine Unlocked*.

[31] Abdoli A. (2020). Iran, sanctions, and the COVID-19 crisis. *Journal of Medical Economics*.

[32] Murphy A., Abdi Z., Harirchi I., McKee M., Ahmadnezhad E. (2020). Economic sanctions and Iran’s capacity to respond to COVID-19. *The Lancet Public Health*.

[33] Han J., Pei J., Kamber M. (2011). *Data mining: concepts and techniques*.

[34] Bhavsar H., Ganatra A. (2012). A comparative study of training algorithms for supervised machine learning. *International Journal of Soft Computing and Engineering*.

[35] Koh H. C., Tan G. (2011). Data mining applications in healthcare. *Journal of Healthcare Information Management*.

[36] Senthilkumar D., Paulraj S. Prediction of low birth weight infants and its risk factors using data mining techniques.

[37] Friedman N., Geiger D., Goldszmidt M. (1997). Bayesian network classifiers. *Machine Learning*.

[38] Cheng J., Greiner R. Comparing Bayesian network classifiers.

[39] Jensen F. V. (1996). *An introduction to Bayesian networks*.

[40] Zhang Z., Chen L., Xu P., Hong Y. (2022). Predictive analytics with ensemble modeling in laparoscopic surgery: a technical note. *Laparoscopic, Endoscopic and Robotic Surgery*.

[41] Seyyed-Kalantari L., Zhang H., McDermott M. B. A., Chen I. Y., Ghassemi M. (2021). Underdiagnosis bias of artificial intelligence algorithms applied to chest radiographs in under-served patient populations. *Nature Medicine*.

[42] Sarkar R., Martin C., Mattie H., Gichoya J. W., Stone D. J., Celi L. A. (2021). Performance of intensive care unit severity scoring systems across different ethnicities in the USA: a retrospective observational study. *The Lancet Digital Health*.

[43] Gao Y., Cai G. Y., Fang W. (2020). Machine learning based early warning system enables accurate mortality risk prediction for COVID-19. *Nature Communications*.

[44] Muhammad L. J., Islam M. M., Usman S. S., Ayon S. I. (2020). Predictive data mining models for novel coronavirus (COVID-19) infected patients’ recovery. *SN Computer Science*.

[45] Booth A. L., Abels E., McCaffrey P. (2020). Development of a prognostic model for mortality in COVID-19 infection using machine learning. *Modern Pathology*.

[46] Barish M., Bolourani S., Lau L. F., Shah S., Zanos T. P. (2021). External validation demonstrates limited clinical utility of the interpretable mortality prediction model for patients with COVID-19. *Nature Machine Intelligence*.

