# Supplementary material for: Machine Learning Models to Predict In-Hospital Mortality among Inpatients with COVID-19: Underestimation and Overestimation Bias Analysis in Subgroup Populations
Source: J Healthc Eng. 2022 Jun 23;2022:1644910. doi: 10.1155/2022/1644910 (PMC9226971; doi:10.1155/2022/1644910)
Supplement: Supplementary Materials — Supplement 1: detailed Tables S1–S14. Supplement 2: Figures S1–S3. [file 1644910.f1.zip › 1644910.f1/supplement1 (1).pdf]

Machine learning models to predict in-hospital mortality among inpatients with COVID-19: Under-estimation and over-estimation bias analysis in subgroup populations

Table S1. The frequency of missing data

**Feature**	**Percentage of missing**
Paresis	3.8
Paralysis	3.8
Skin Manifestations	3.8
Vomiting	3.1
Chest Pain	2.6
Dizziness	2.6
Headache	2.6
Abdominal Pain	2.0
Diarrhea	1.9
Nausea	1.9
Anorexia	1.9
Gustatory Dysfunction	1.2
Convulsion	1.2
Other Diseases(Comorbidities)	1.2
Drug Abuse	1.2
Olfactory Dysfunction	1.2
Sweating	1.2
Asthma	1.2
Constipation	1.2
GI bleeding	1.2
Other GI Signs	1.2
Blood Oxygen Saturation Level	1.2
Smoke	1.2
Mental disorder	1.2
Ocular Manifestations	1.1
Age	0.9
ICU admission	0.7
Intubation	0.7

Table S2. The models developed on the original dataset 1 and the feature set 1 (test data)

**Model**	**Settings**	**Dataset-Feature set**	**Accuracy**	**Sensitivity**	**Specificity**	**Precision**	**F score**	**AUC**
RF	Default	1-1	84.62	0.728	0.869	0.513	0.602	0.87
C5	Default	1-1	90.41	0.506	0.980	0.827	0.628	0.762
C5	Boosting	1-1	90.41	0.506	0.980	0.827	0.628	0.762
CHIAD	Default	1-1	90.41	0.506	0.980	0.827	0.628	0.896
CHIAD	Boosting	1-1	90.41	0.506	0.980	0.827	0.628	0.896
CHIAD exhaustive	Default	1-1	90.41	0.506	0.980	0.827	0.628	0.896
CHIAD exhaustive	Boosting	1-1	90.09	0.554	0.967	0.761	0.641	0.891
SVM	RBF Default	1-1	90.28	0.528	0.974	0.795	0.635	0.843
Bayesian network	Default	1-1	90.46	0.555	0.971	0.785	0.650	0.899
MLP *	Default 1.8	1-1	90.15	0.546	0.969	0.771	0.639	0.894
MLP	1.20	1-1	90.38	0.520	0.977	0.810	0.633	0.891
MLP	1.10	1-1	90.41	0.526	0.976	0.807	0.637	0.888
MLP	1.5	1-1	90.38	0.504	0.980	0.826	0.626	0.877
MLP	2.10.10	1-1	90.03	0.526	0.971	0.778	0.628	0.896
MLP	2.20.20	1-1	90.41	0.530	0.975	0.803	0.639	0.886
MLP	2.8.8	1-1	90.60	0.540	0.976	0.808	0.647	0.888
MLP	2.5.5	1-1	90.31	0.554	0.970	0.776	0.646	0.901
MLP	1.8 Boosting	1-1	89.00	0.556	0.954	0.695	0.618	0.844
MLP	1.15	1-1	90.22	0.552	0.969	0.771	0.643	0.886
MLP	2.5.5 Boosting	1-1	90.63	0.536	0.977	0.815	0.647	0.904
MLP	2.15.15	1-1	90.19	0.522	0.974	0.793	0.630	0.885

* For MLPs, the default setting indicates the network structure suggested by the software. The numbers for MLP indicate the number of layers, the number of neurons in the hidden layer 1 and the number of neurons in the hidden layer 2.

Table S3. The models developed on the original dataset 1 and the feature set 2 (test data)

**Model**	**Settings**	**Dataset-Feature set**	**Accuracy**	**Sensitivity**	**Specificity**	**Precision**	**F score**	**AUC**
RF	Default	1-2	84.52	0.775	0.859	0.513	0.617	0.9
C5	Default	1-2	89.83	0.550	0.965	0.747	0.634	0.757
C5	Boosting	1-2	90.70	0.564	0.973	0.799	0.662	0.901
CHIAD	Default	1-2	90.76	0.540	0.978	0.826	0.653	0.909
CHIAD	Boosting	1-2	89.96	0.564	0.963	0.746	0.642	0.881
CHIAD exhaustive	Default	1-2	90.19	0.522	0.974	0.793	0.630	0.893
CHIAD exhaustive	Boosting	1-2	90.19	0.522	0.974	0.793	0.630	0.893
SVM	RBF Default	1-2	90.64	0.581	0.969	0.782	0.667	0.879
Bayesian network	Default	1-2	91.12	0.647	0.962	0.764	0.701	0.914
MLP*	Default 1.11	1-2	90.35	0.548	0.971	0.783	0.645	0.889
MLP	1.20	1-2	90.25	0.522	0.975	0.798	0.631	0.881
MLP	1.10	1-2	90.12	0.530	0.972	0.782	0.632	0.88
MLP	1.5	1-2	90.25	0.518	0.976	0.802	0.629	0.885
MLP	2.10.10	1-2	90.55	0.534	0.977	0.815	0.646	0.901
MLP	2.20.20	1-2	89.93	0.488	0.978	0.805	0.608	0.877
MLP	2.11.11	1-2	90.44	0.546	0.973	0.791	0.646	0.894
MLP	2.5.5	1-2	90.12	0.540	0.970	0.774	0.636	0.896
MLP	2.10.10 Boosting	1-2	88.87	0.570	0.949	0.682	0.621	0.871
MLP	1.15	1-2	90.19	0.532	0.972	0.785	0.634	0.882
MLP	2.11.11 Boosting	1-2	88.14	0.540	0.946	0.657	0.593	0.863
MLP	2.15.15	1-2	90.31	0.524	0.975	0.801	0.634	0.889

* For MLPs, the default setting indicates the network structure suggested by the software. The numbers for MLP indicate the number of layers, the number of neurons in the hidden layer 1 and the number of neurons in the hidden layer 2.

Table S4. The models developed on the original dataset 1 and the feature set 3(test data)

**Model**	**Settings**	**Dataset-Feature set**	**Accuracy**	**Sensitivity**	**Specificity**	**Precision**	**F score**	**AUC**
RF	Default	1-3	85.17	0.746	0.872	0.525	0.617	0.882
C5	Default	1-3	90.57	0.542	0.975	0.804	0.648	0.835
C5	Boosting	1-3	90.70	0.538	0.977	0.818	0.649	0.885
CHIAD	Default	1-3	90.41	0.506	0.980	0.827	0.628	0.896
CHIAD	Boosting	1-3	90.41	0.506	0.980	0.827	0.628	0.896
CHIAD exhaustive	Default	1-3	90.41	0.506	0.980	0.827	0.628	0.896
CHIAD exhaustive	Boosting	1-3	90.09	0.554	0.967	0.761	0.641	0.891
SVM	RBF Default	1-3	89.71	0.584	0.957	0.719	0.645	0.852
Bayesian network	Default	1-3	90.90	0.586	0.970	0.789	0.673	0.893
MLP*	Default 1.12	1-3	90.44	0.556	0.971	0.783	0.650	0.887
MLP	1.20	1-3	90.31	0.550	0.970	0.779	0.645	0.891
MLP	1.10	1-3	90.35	0.514	0.978	0.813	0.630	0.89
MLP	1.5	1-3	90.19	0.550	0.969	0.770	0.642	0.889
MLP	2.10.10	1-3	89.99	0.540	0.968	0.765	0.633	0.891
MLP	2.20.20	1-3	90.51	0.536	0.975	0.805	0.643	0.899
MLP	2.12.12	1-3	90.66	0.516	0.981	0.838	0.639	0.869
MLP	2.5.5	1-3	90.41	0.528	0.976	0.805	0.638	0.891
MLP	1.12 Boosting	1-3	88.24	0.598	0.936	0.642	0.619	0.879
MLP	1.15	1-3	90.47	0.524	0.977	0.814	0.637	0.869
MLP	1.10 Boosting	1-3	90.79	0.540	0.978	0.823	0.652	0.903
MLP	2.15.15	1-3	90.51	0.536	0.975	0.805	0.643	0.87

* For MLPs, the default setting indicates the network structure suggested by the software. The numbers for MLP indicate the number of layers, the number of neurons in the hidden layer 1 and the number of neurons in the hidden layer 2.

Table S5. The models developed on the original dataset 1 and the feature set 4 (test data)

**Model**	**Settings**	**Dataset-Feature set**	**Accuracy**	**Sensitivity**	**Specificity**	**Precision**	**F score**	**AUC**
RF	Default	1-4	86.60	0.728	0.892	0.563	0.635	0.874
C5	Default	1-4	90.47	0.522	0.978	0.816	0.637	0.761
C5	Boosting	1-4	90.60	0.574	0.969	0.780	0.661	0.886
CHIAD	Default	1-4	90.41	0.506	0.980	0.827	0.628	0.896
CHIAD	Boosting	1-4	89.64	0.524	0.967	0.753	0.618	0.894
CHIAD exhaustive	Default	1-4	90.41	0.506	0.980	0.827	0.628	0.897
CHIAD exhaustive	Boosting	1-4	90.41	0.506	0.980	0.827	0.628	0.897
SVM	RBF Default	1-4	89.26	0.606	0.947	0.686	0.643	0.861
Bayesian network	Default	1-4	90.64	0.578	0.969	0.778	0.664	0.889
MLP*	Default 1.12	1-4	90.22	0.550	0.969	0.772	0.643	0.895
MLP	1.20	1-4	90.06	0.496	0.978	0.808	0.615	0.872
MLP	1.10	1-4	90.63	0.584	0.968	0.775	0.666	0.897
MLP	1.5	1-4	90.44	0.520	0.978	0.815	0.635	0.887
MLP	2.10.10	1-4	90.06	0.508	0.975	0.796	0.620	0.876
MLP	2.20.20	1-4	90.47	0.522	0.978	0.816	0.637	0.883
MLP	2.12.12	1-4	89.99	0.522	0.972	0.779	0.625	0.88
MLP	2.5.5	1-4	90.19	0.530	0.973	0.786	0.633	0.89
MLP	1.10 Boosting	1-4	90.12	0.558	0.967	0.760	0.644	0.898
MLP	1.15	1-4	90.47	0.536	0.975	0.802	0.643	0.895
MLP	1.15 Boosting	1-4	87.98	0.544	0.944	0.648	0.591	0.872
MLP	2.15.15	1-4	90.12	0.484	0.981	0.826	0.610	0.869

* For MLPs, the default setting indicates the network structure suggested by the software. The numbers for MLP indicate the number of layers, the number of neurons in the hidden layer 1 and the number of neurons in the hidden layer 2.

Table S6. The models developed on the dataset 2 and the feature set 1 (test data)

Model	Settings	Dataset-Feature set	Accuracy	Sensitivity	Specificity	Precision	F score	AUC
RF	Default	2-1	85.26	0.852	0.853	0.762	0.804	0.923
C5	Default	2-1	85.33	0.792	0.887	0.794	0.793	0.909
C5	Boosting	2-1	85.99	0.772	0.908	0.822	0.797	0.926
CHIAD	Default	2-1	84.11	0.765	0.883	0.783	0.774	0.915
CHIAD	Boosting	2-1	83.80	0.772	0.875	0.772	0.772	0.913
CHIAD exhaustive	Default	2-1	84.67	0.769	0.889	0.793	0.781	0.917
CHIAD exhaustive	Boosting	2-1	84.51	0.764	0.889	0.792	0.778	0.914
SVM	RBF Default	2-1	85.94	0.770	0.908	0.823	0.796	0.922
Bayesian network	Default	2-1	84.36	0.739	0.901	0.805	0.771	0.917
MLP*	Default 1.6	2-1	85.30	0.760	0.904	0.814	0.786	0.919
MLP	1.20	2-1	84.81	0.724	0.916	0.827	0.772	0.915
MLP	1.10	2-1	85.35	0.754	0.908	0.819	0.785	0.919
MLP	1.5	2-1	85.66	0.747	0.917	0.832	0.787	0.92
MLP	2.10.10	2-1	85.61	0.755	0.912	0.825	0.789	0.92
MLP	2.20.20	2-1	85.33	0.752	0.909	0.820	0.785	0.916
MLP	2.6.6	2-1	85.47	0.756	0.909	0.821	0.787	0.921
MLP	2.5.5	2-1	85.70	0.760	0.911	0.824	0.791	0.92
MLP	1.5 Boosting	2-1	85.54	0.756	0.910	0.823	0.788	0.92
MLP	1.15	2-1	85.52	0.743	0.917	0.832	0.785	0.919
MLP	2.5.5 Boosting	2-1	85.21	0.760	0.903	0.811	0.785	0.921
MLP	2.15.15	2-1	85.63	0.760	0.910	0.822	0.790	0.921

* For MLPs, the default setting indicates the network structure suggested by the software. The numbers for MLP indicate the number of layers, the number of neurons in the hidden layer 1 and the number of neurons in the hidden layer 2.

Table S7. The models developed on the dataset 2 and the feature set 2 (test data)

Model	Settings	Dataset-Feature set	Accuracy	Sensitivity	Specificity	Precision	F score	AUC
RF	Default	2-2	85.99	0.839	0.872	0.793	0.815	0.911
C5	Default	2-2	85.11	0.794	0.883	0.793	0.793	0.894
C5	Boosting	2-2	86.68	0.785	0.915	0.843	0.813	0.927
CHIAD	Default	2-2	83.94	0.658	0.945	0.875	0.751	0.907
CHIAD	Boosting	2-2	83.59	0.724	0.899	0.801	0.760	0.901
CHIAD exhaustive	Default	2-2	83.85	0.711	0.910	0.816	0.760	0.901
CHIAD exhaustive	Boosting	2-2	83.50	0.724	0.898	0.799	0.759	0.898
SVM	RBF Default	2-2	86.61	0.790	0.911	0.837	0.813	0.926
Bayesian network	Default	2-2	84.79	0.727	0.918	0.838	0.779	0.912
MLP*	Default 1.11	2-2	84.33	0.719	0.913	0.823	0.767	0.906
MLP	1.20	2-2	84.92	0.724	0.919	0.835	0.775	0.908
MLP	1.10	2-2	84.66	0.715	0.921	0.835	0.770	0.907
MLP	1.5	2-2	84.47	0.715	0.918	0.830	0.768	0.905
MLP	2.10.10	2-2	84.45	0.720	0.915	0.825	0.769	0.908
MLP	2.20.20	2-2	84.66	0.708	0.924	0.840	0.769	0.905
MLP	2.11.11	2-2	84.80	0.729	0.915	0.827	0.775	0.905
MLP	2.5.5	2-2	83.85	0.714	0.908	0.814	0.761	0.898
MLP	1.10 Boosting	2-2	84.40	0.758	0.894	0.807	0.782	0.911
MLP	1.15	2-2	84.33	0.720	0.912	0.822	0.768	0.906
MLP	1.20 Boosting	2-2	84.64	0.720	0.917	0.830	0.771	0.911
MLP	2.15.15	2-2	84.99	0.710	0.928	0.848	0.773	0.904

* For MLPs, the default setting indicates the network structure suggested by the software. The numbers for MLP indicate the number of layers, the number of neurons in the hidden layer 1 and the number of neurons in the hidden layer 2.

Table S8. The models developed on the dataset 2 and the feature set 3 (test data)

**Model**	**Settings**	**Dataset-Feature set**	**Accuracy**	**Sensitivity**	**Specificity**	**Precision**	**F score**	**AUC**
RF	Default	2-3	85.23	0.861	0.848	0.757	0.806	0.908
C5	Default	2-3	85.61	0.792	0.891	0.801	0.796	0.918
C5	Boosting	2-3	87.44	0.818	0.906	0.827	0.822	0.94
CHIAD	Default	2-3	84.11	0.765	0.883	0.783	0.774	0.915
CHIAD	Boosting	2-3	84.65	0.765	0.891	0.795	0.780	0.915
CHIAD exhaustive	Default	2-3	84.67	0.769	0.889	0.793	0.781	0.917
CHIAD exhaustive	Boosting	2-3	85.05	0.790	0.884	0.790	0.790	0.92
SVM	RBF Default	2-3	87.59	0.827	0.903	0.824	0.826	0.938
Bayesian network	Default	2-3	85.23	0.756	0.906	0.815	0.784	0.921
MLP*	Default 1.10	2-3	85.38	0.770	0.900	0.809	0.789	0.923
MLP	1.20	2-3	85.77	0.735	0.926	0.845	0.786	0.922
MLP	1.5	2-3	85.82	0.763	0.911	0.825	0.793	0.919
MLP	2.10.10	2-3	85.38	0.758	0.907	0.817	0.786	0.919
MLP	2.20.20	2-3	85.70	0.780	0.900	0.811	0.795	0.922
MLP	2.5.5	2-3	85.56	0.768	0.904	0.815	0.791	0.918
MLP	1.10 Boosting	2-3	85.82	0.775	0.904	0.817	0.795	0.920
MLP	1.15	2-3	86.39	0.775	0.913	0.830	0.802	0.919
MLP	1.15 Boosting	2-3	84.15	0.788	0.871	0.771	0.779	0.915
MLP	2.15.15	2-3	85.77	0.762	0.910	0.824	0.792	0.919

* For MLPs, the default setting indicates the network structure suggested by the software. The numbers for MLP indicate the number of layers, the number of neurons in the hidden layer 1 and the number of neurons in the hidden layer 2.

Table S9. The models developed on the dataset 2 and the feature set 4 (test data)

**Model**	**Settings**	**Dataset-Feature set**	**Accuracy**	**Sensitivity**	**Specificity**	**Precision**	**F score**	**AUC**
RF	Default	2-4	87.86	0.857	0.891	0.815	0.836	0.931
C5	Default	2-4	85.86	0.789	0.898	0.813	0.801	0.905
C5	Boosting	2-4	87.88	0.799	0.924	0.855	0.826	0.938
CHIAD	Default	2-4	83.70	0.671	0.930	0.844	0.748	0.904
CHIAD	Boosting	2-4	83.72	0.716	0.906	0.810	0.760	0.904
CHIAD exhaustive	Default	2-4	83.70	0.671	0.930	0.844	0.748	0.904
CHIAD exhaustive	Boosting	2-4	83.68	0.705	0.911	0.817	0.757	0.901
SVM	RBF Default	2-4	87.83	0.834	0.903	0.829	0.832	0.942
Bayesian network	Default	2-4	83.70	0.714	0.906	0.811	0.759	0.904
MLP*	Default 1.11	2-4	83.21	0.731	0.889	0.788	0.758	0.899
MLP	1.20	2-4	82.64	0.684	0.907	0.805	0.739	0.888
MLP	1.10	2-4	84.88	0.731	0.915	0.829	0.777	0.908
MLP	1.5	2-4	83.51	0.693	0.915	0.821	0.752	0.894
MLP	2.10.10	2-4	83.94	0.729	0.902	0.806	0.766	0.9
MLP	2.20.20	2-4	83.49	0.707	0.907	0.810	0.755	0.897
MLP	2.11.11	2-4	82-3	0.691	0.914	0.819	0.750	0.896
MLP	2.5.5	2-4	84.48	0.738	0.905	0.814	0.774	0.908
MLP	1.10 Boosting	2-4	84.69	0.720	0.919	0.832	0.772	0.906
MLP	1.15	2-4	84.55	0.719	0.917	0.829	0.770	0.908
MLP	1.15 Boosting	2-4	84.57	0.749	0.900	0.808	0.778	0.908
MLP	2.15.15	2-4	83.70	0.697	0.916	0.823	0.755	0.901

* For MLPs, the default setting indicates the network structure suggested by the software. The numbers for MLP indicate the number of layers, the number of neurons in the hidden layer 1 and the number of neurons in the hidden layer 2.

Table S10. The models developed on the dataset 3 and the feature set 1 (test data)

Model	Settings	Dataset-Feature set	Accuracy	Sensitivity	Specificity	Precision	F score	AUC
RF †	Default	3-1	86.12	0.841	0.882	0.876	0.858	0.923
C5	Default	3-1	85.41	0.866	0.843	0.845	0.855	0.925
C5 †	Boosting	3-1	89.28	0.913	0.873	0.877	0.895	0.952
CHIAD	Default	3-1	82.78	0.810	0.846	0.839	0.824	0.912
CHIAD †	Boosting	3-1	83.12	0.808	0.855	0.847	0.827	0.914
CHIAD exhaustive	Default	3-1	82.78	0.810	0.846	0.839	0.824	0.912
CHIAD exhaustive	Boosting	3-1	82.73	0.796	0.858	0.848	0.821	0.912
SVM †	RBF Default	3-1	85.33	0.852	0.854	0.853	0.853	0.927
Bayesian network †	Default	3-1	81.99	0.797	0.843	0.835	0.815	0.909
MLP*	Default 1.7	3-1	82.93	0.803	0.855	0.847	0.824	0.915
MLP	1.20	3-1	82.35	0.781	0.866	0.853	0.815	0.912
MLP	1.10	3-1	82.59	0.783	0.868	0.855	0.818	0.915
MLP	1.5	3-1	82.97	0.801	0.858	0.849	0.824	0.916
MLP	2.10.10	3-1	82.59	0.787	0.865	0.853	0.819	0.915
MLP	2.20.20	3-1	83.24	0.821	0.843	0.839	0.830	0.915
MLP	2.7.7	3-1	82.78	0.791	0.864	0.853	0.821	0.913
MLP	2.5.5	3-1	83.22	0.802	0.862	0.852	0.827	0.915
MLP †	1.5 Boosting	3-1	83.07	0.799	0.862	0.852	0.825	0.916
MLP	1.15	3-1	82.63	0.792	0.861	0.850	0.820	0.917
MLP	2.5.5 Boosting	3-1	82.86	0.802	0.855	0.847	0.823	0.919
MLP	2.15.15	3-1	83.42	0.810	0.858	0.851	0.830	0.914
Ensemble	-	3-1	86.10	0.799	0.924	0.914	0.853	0.954
C5	Boosting	3-1	89.28	0.913	0.873	0.877	0.895	0.952

* For MLPs, the default setting indicates the network structure suggested by the software. The numbers for MLP indicate the number of layers, the number of neurons in the hidden layer 1 and the number of neurons in the hidden layer 2.

† These models were used to develop ensemble models.

Table S11. The models developed on the dataset 3 and the feature set 2 (test data)

Model	Settings	Dataset-Feature set	Accuracy	Sensitivity	Specificity	Precision	F score	AUC
RF †	Default	3-2	85.11	0.841	0.862	0.863	0.852	0.907
C5	Default	3-2	85.85	0.886	0.831	0.839	0.862	0.926
C5 †	Boosting	3-2	91.18	0.942	0.880	0.891	0.916	0.96
CHIAD	Default	3-2	81.27	0.783	0.842	0.831	0.806	0.9
CHIAD	Boosting	3-2	81.59	0.806	0.826	0.821	0.813	0.899
CHIAD exhaustive	Default	3-2	81.27	0.783	0.842	0.831	0.806	0.9
CHIAD exhaustive †	Boosting	3-2	82.29	0.816	0.830	0.832	0.824	0.906
SVM †	RBF Default	3-2	86.34	0.866	0.861	0.866	0.866	0.932
Bayesian network †	Default	3-2	82.15	0.792	0.852	0.848	0.819	0.906
MLP*	Default 1.9	3-2	82.11	0.782	0.860	0.847	0.813	0.908
MLP	1.20	3-2	81.98	0.786	0.853	0.842	0.813	0.906
MLP	1.10	3-2	82.00	0.782	0.857	0.845	0.812	0.906
MLP	1.5	3-2	81.59	0.783	0.848	0.837	0.809	0.902
MLP	2.10.10	3-2	82.24	0.799	0.845	0.837	0.818	0.909
MLP	2.20.20	3-2	81.33	0.786	0.841	0.830	0.807	0.906
MLP	2.9.9	3-2	82.24	0.794	0.850	0.840	0.817	0.905
MLP	2.5.5	3-2	81.76	0.783	0.851	0.840	0.811	0.907
MLP	1.9 Boosting	3-2	82.15	0.782	0.860	0.847	0.814	0.912
MLP	1.15	3-2	81.70	0.768	0.866	0.850	0.807	0.903
MLP †	1.10 Boosting	3-2	83.48	0.811	0.860	0.857	0.833	0.913
MLP	1.15.15	3-2	82.05	0.794	0.847	0.838	0.815	0.905
Ensemble		3-2	87.39	0.859	0.889	0.888	0.873	0.954

* For MLPs, the default setting indicates the network structure suggested by the software. The numbers for MLP indicate the number of layers, the number of neurons in the hidden layer 1 and the number of neurons in the hidden layer 2.

† These models were used to develop ensemble models.

Table S12. The models developed on the dataset 3 and the feature set 3 (test data)

**Model**	**Settings**	**Dataset-Feature set**	**Accuracy**	**Sensitivity**	**Specificity**	**Precision**	**F score**	**AUC**
RF†	Default	3-3	86.43	0.850	0.880	0.879	0.864	0.917
C5	Default	3-3	87.40	0.898	0.850	0.861	0.879	0.934
C5†	Boosting	3-3	91.74	0.936	0.898	0.905	0.920	0.965
CHIAD	Default	3-3	81.50	0.797	0.834	0.832	0.814	0.904
CHIAD†	Boosting	3-3	83.16	0.823	0.840	0.842	0.833	0.911
CHIAD exhaustive	Default	3-3	81.59	0.803	0.830	0.830	0.816	0.905
CHIAD exhaustive	Boosting	3-3	83.12	0.813	0.850	0.849	0.830	0.911
SVM †	RBF Default	3-3	88.81	0.905	0.871	0.879	0.892	0.944
Bayesian network†	Default	3-3	82.91	0.802	0.858	0.853	0.827	0.91
MLP*	Default 1.11	3-3	83.58	0.825	0.847	0.848	0.836	0.915
MLP	1.20	3-3	82.56	0.802	0.850	0.847	0.824	0.91
MLP	1.10	3-3	82.97	0.787	0.874	0.866	0.824	0.911
MLP	1.5	3-3	83.47	0.811	0.859	0.856	0.833	0.912
MLP	2.10.10	3-3	83.47	0.795	0.875	0.869	0.830	0.909
MLP	2.20.20	3-3	82.52	0.785	0.867	0.859	0.820	0.901
MLP	2.11.11	3-3	82.54	0.801	0.851	0.848	0.823	0.906
MLP	2.5.5	3-3	83.79	0.820	0.856	0.855	0.837	0.912
MLP	2.5.5 Boosting	3-3	84.20	0.828	0.857	0.857	0.842	0.918
MLP	1.15	3-3	82-2	0.798	0.869	0.863	0.830	0.909
MLP	1.11 Boosting	3-3	86.66	0.890	0.842	0.854	0.872	0.923
MLP	2.15.15	3-3	83.77	0.805	0.872	0.866	0.835	0.913
MLP†	2.15.15 Boosting	3-3	88.59	0.902	0.869	0.877	0.889	0.94
Ensemble			87.26	0.831	0.915	0.908	0.867	0.954

* For MLPs, the default setting indicates the network structure suggested by the software. The numbers for MLP indicate the number of layers, the number of neurons in the hidden layer 1 and the number of neurons in the hidden layer 2.

† These models were used to develop ensemble models.

Table S13. The models developed on the dataset 3 and the feature set 4 (test data)

Model	Settings	Dataset-Feature set	Accuracy	Sensitivity	Specificity	Precision	F score	AUC
RF†	Default	3-4	85.85	0.850	0.867	0.863	0.856	0.927
C5	Default	3-4	87.78	0.892	0.864	0.866	0.879	0.931
C5†	Boosting	3-4	92.77	0.951	0.905	0.908	0.929	0.972
CHIAD	Default	3-4	83.10	0.806	0.855	0.846	0.826	0.909
CHIAD	Boosting	3-4	83.47	0.813	0.856	0.848	0.830	0.911
CHIAD exhaustive	Default	3-4	82.95	0.819	0.840	0.834	0.827	0.91
CHIAD exhaustive †	Boosting	3-4	83.74	0.825	0.849	0.844	0.834	0.913
SVM †	RBF Default	3-4	90.16	0.927	0.877	0.881	0.903	0.956
Bayesian network†	Default	3-4	82.52	82.69	0.800	0.853	0.843	0.911
MLP*	Default 1.12	3-4	82.05	81.17	0.778	0.845	0.832	0.9
MLP	1.20	3-4	82.11	0.795	0.847	0.837	0.815	0.908
MLP	1.10	3-4	82.54	0.793	0.857	0.845	0.818	0.911
MLP	1.5	3-4	82.09	0.783	0.858	0.845	0.813	0.906
MLP	2.10.10	3-4	82.07	0.794	0.847	0.836	0.815	0.901
MLP	2.20.20	3-4	82.86	0.796	0.860	0.849	0.822	0.909
MLP	2.12.12	3-4	82.71	0.804	0.850	0.841	0.822	0.902
MLP	2.5.5	3-4	82.69	0.807	0.847	0.838	0.822	0.909
MLP	1.15	3-4	81.97	0.752	0.887	0.867	0.805	0.906
MLP	2.15.15	3-4	82.46	0.801	0.848	0.839	0.819	0.908
MLP†	2.12.12 Boosting	3-4	87.61	0.885	0.868	0.868	0.876	0.938
Ensemble		3-4	89.13	0.864	0.919	0.916	0.890	0.961

* For MLPs, the default setting indicates the network structure suggested by the software. The numbers for MLP indicate the number of layers, the number of neurons in the hidden layer 1 and the number of neurons in the hidden layer 2.

† These models were used to develop ensemble models.

Table S14: Subgroup bias analysis

Models	Subgroup	Accuracy	Sensitivity	Specificity	Precision	F score	FNR	FPR	AUC
C5 Boosting (Feature set 2, Dataset 3)	Male	90.82	0.967	0.893	0.696	0.809	0.03	0.11	0.974
	Female	92.27	0.946	0.919	0.678	0.790	0.05	0.08	0.974
	0-20 years	93.33	1	0.926	0.600	0.750	0.00	0.07	1
	21-40 years	96.88	1	0.967	0.630	0.773	0.00	0.03	0.992
	41-60 years	94.71	0.912	0.952	0.705	0.795	0.09	0.05	0.976
	61-80 years	87.22	0.955	0.844	0.674	0.790	0.04	0.16	0.962
	81+ years	85.06	1	0.747	0.733	0.846	0.00	0.25	0.954
C 5 Boosting (Feature set 1, Dataset 3)	Male	88.91	0.944	0.875	0.656	0.774	0.06	0.12	0.963
	Female	93.10	0.953	0.927	0.703	0.809	0.05	0.07	0.979
	0-20 years	93.33	0.667	0.963	0.667	0.667	0.33	0.04	0.901
	21-40 years	95.63	0.941	0.957	0.552	0.696	0.06	0.04	0.982
	41-60 years	93.72	0.897	0.942	0.663	0.763	0.10	0.06	0.97
	61-80 years	87.38	0.955	0.846	0.677	0.793	0.04	0.15	0.969
	81+ years	84.42	1	0.736	0.724	0.840	0.00	0.26	0.945
SVM RBF Default (Feature set 3, Dataset 3)	Male	89.81	0.856	0.909	0.703	0.772	0.14	0.09	0.952
	Female	92.27	0.837	0.938	0.711	0.769	0.16	0.06	0.957
	0-20 years	100.0	1	1	1	1	0.00	0.00	1
	21-40 years	96.25	1	0.960	0.586	0.739	0.00	0.04	0.994
	41-60 years	92.56	0.779	0.944	0.639	0.702	0.22	0.06	0.94
	61-80 years	89.00	0.872	0.896	0.739	0.800	0.13	0.10	0.941
	81+ years	80.52	0.810	0.802	0.739	0.773	0.19	0.20	0.912
Ensemble 2 (Feature set 2, Dataset 3)	Male	86.23	0.883	0.857	0.609	0.721	0.12	0.14	0.95
	Female	89.54	0.853	0.903	0.615	0.714	0.15	0.10	0.956
	0-20 years	93.33	0.667	0.963	0.667	0.667	0.33	0.04	0.926
	21-40 years	95.63	0.882	0.960	0.556	0.682	0.12	0.04	0.986
	41-60 years	91.07	0.765	0.929	0.578	0.658	0.24	0.07	0.949
	61-80 years	83.98	0.904	0.818	0.627	0.740	0.10	0.18	0.941
	81+ years	73.38	0.905	0.615	0.620	0.735	0.10	0.38	0.905
