# Supplementary material for: Machine Learning Models to Predict In-Hospital Mortality among Inpatients with COVID-19: Underestimation and Overestimation Bias Analysis in Subgroup Populations
Source: J Healthc Eng. 2022 Jun 23;2022:1644910. doi: 10.1155/2022/1644910 (PMC9226971; doi:10.1155/2022/1644910)
Supplement: Supplementary Materials — Supplement 1: detailed Tables S1–S14. Supplement 2: Figures S1–S3. [file 1644910.f1.zip › 1644910.f1/supplement2.pdf]

Machine learning models to predict in-hospital mortality among inpatients with COVID-19: Under-estimation and over-estimation bias analysis in subgroup populations

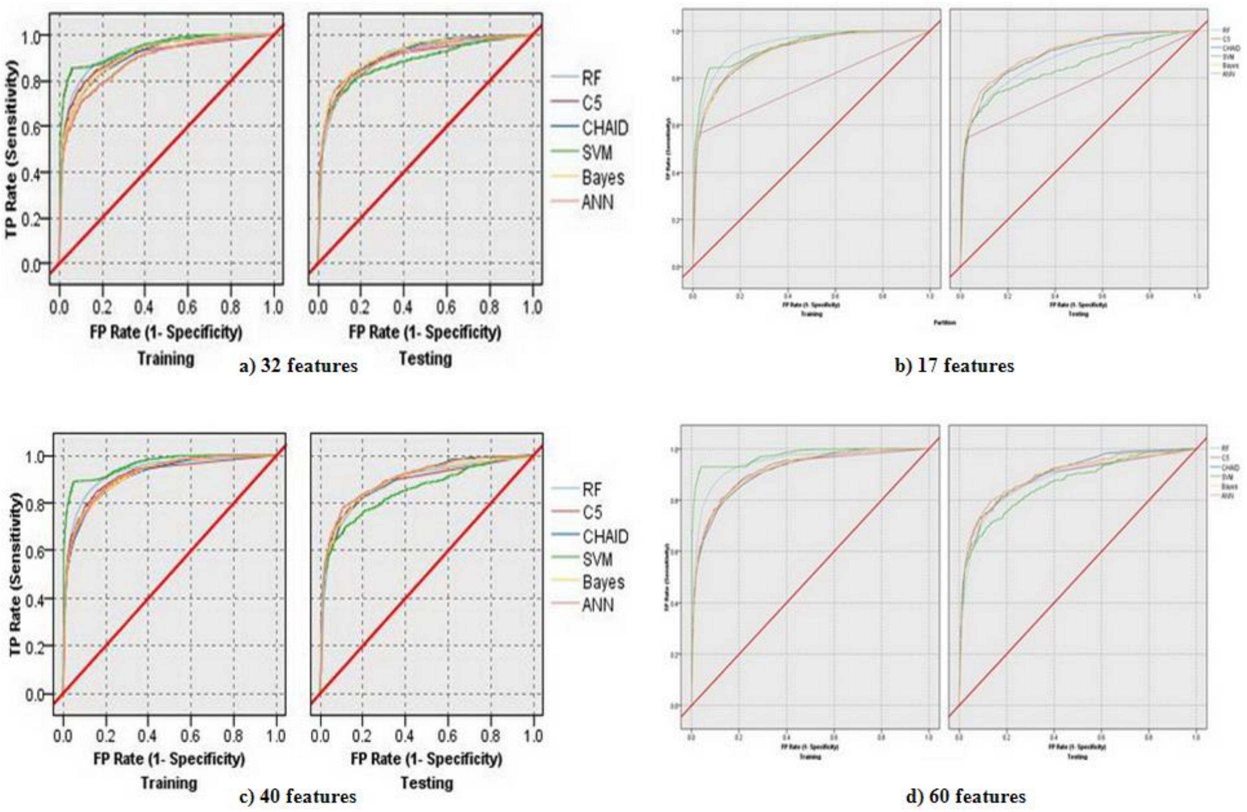

Figure S1. ROC curve for the best results obtained for each algorithm based on the original dataset 1 and different feature sets

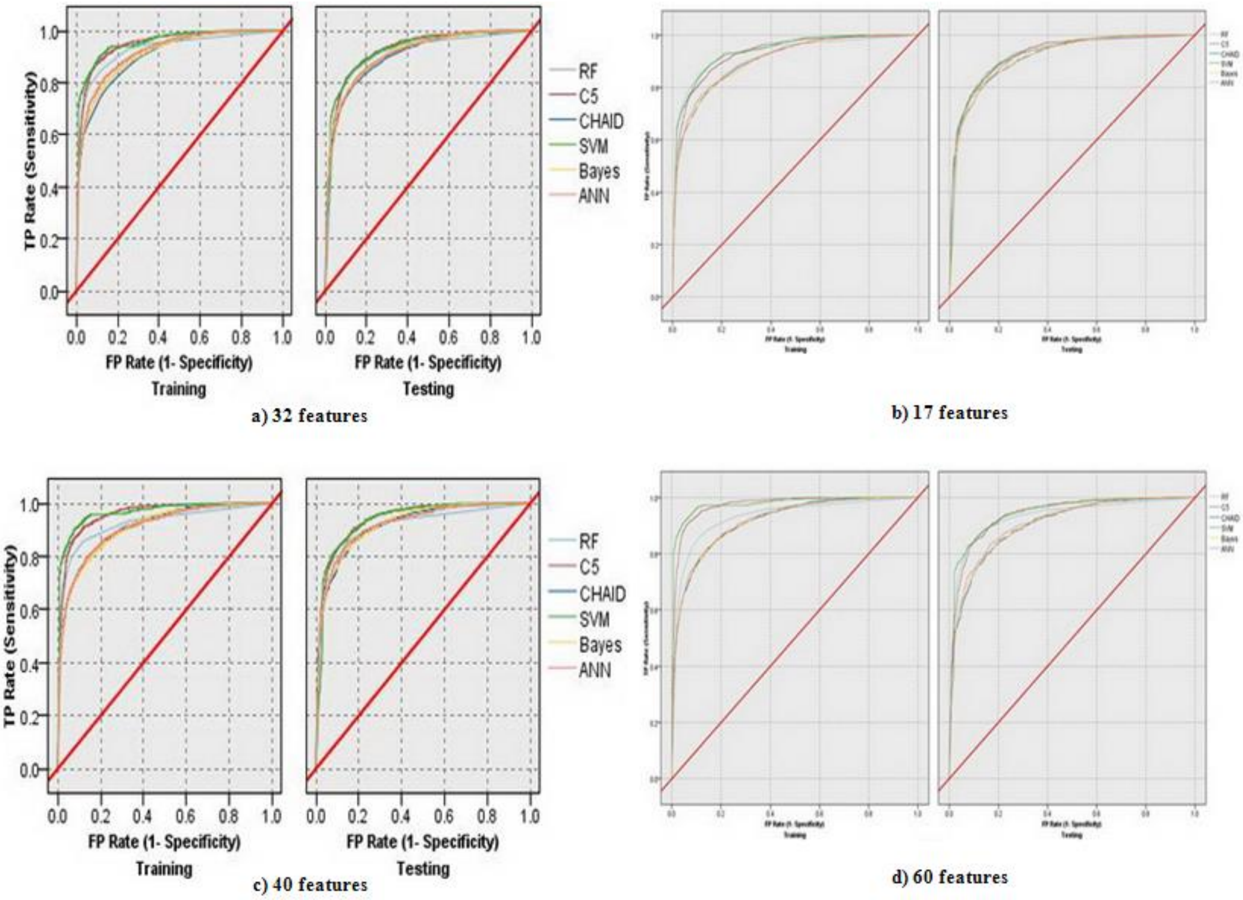

Figure S2. ROC curve for the best results obtained for each algorithm based on dataset 2 and different feature sets

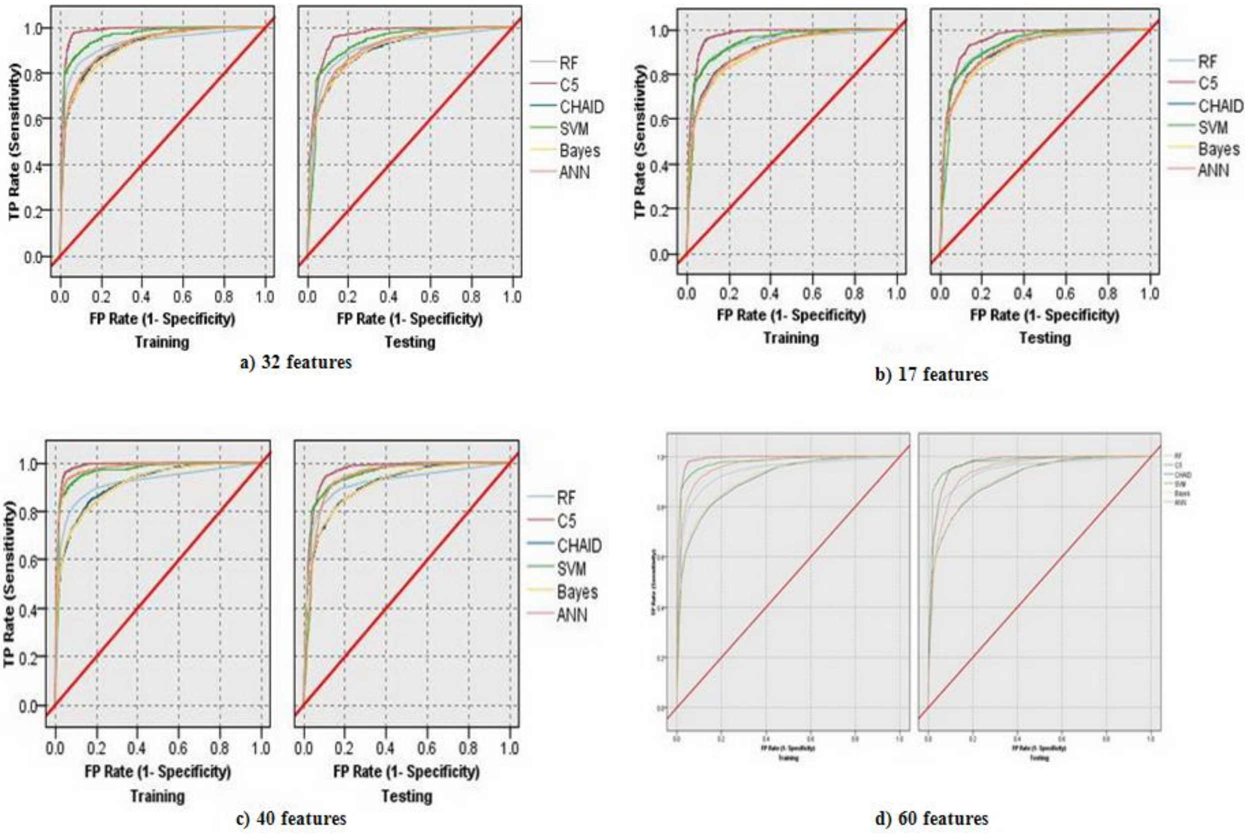

Figure S3. ROC curve for the best results obtained for each algorithm based on dataset 3 and different feature sets
